# Effects of Functional Training on Sarcopenia in Elderly Women in the Presence or Absence of ACE Inhibitors

**DOI:** 10.3390/ijerph18126594

**Published:** 2021-06-19

**Authors:** Marianna Mile, László Balogh, Gábor Papp, József Márton Pucsok, Krisztina Szabó, Lilla Barna, Zoltán Csiki, István Lekli

**Affiliations:** 1 Institute of Sport Sciences, University of Debrecen, 4032 Debrecen, Hungary; marianna.mile@gmail.com (M.M.); balogh.laszlo@sport.unideb.hu (L.B.); papp.gabor@med.unideb.hu (G.P.); pucsok.jozsef@sport.unideb.hu (J.M.P.); barna.lilla@sport.unideb.hu (L.B.); 2 Department of Internal Medicine, Faculty of Medicine, University of Debrecen, 4032 Debrecen, Hungary; krisztinaszabo@med.unideb.hu (K.S.); csiki@med.unideb.hu (Z.C.); 3 Institute of Healthcare Industry, University of Debrecen, 4032 Debrecen, Hungary; 4 Department of Pharmacology, Faculty of Pharmacy, University of Debrecen, 4032 Debrecen, Hungary

**Keywords:** sarcopenia, elderly, ACE inhibitors, functional training

## Abstract

Sarcopenia, defined as loss of muscle mass and strength, develops gradually with aging or after chronic disease. Efforts are ongoing to identify the best interventions that can slow down or stop sarcopenia. Nutrition-based interventions and exercise therapy may be beneficial; however, pharmacotherapy also could play a role. The effect of ACE inhibitors on physical performance is controversial. The present study investigates the impact of functional training on sarcopenia in the presence or absence of ACEi in elderly females. A total of 35 women over 65 years of age were selected for two groups on the basis that they were taking ACEi (*n* = 18) or not (*n* = 17). All subjects conducted a training program two times a week for 6 months. We examined various factors related to sarcopenia. After completing the short physical performance battery (SPPB) test, we found a significant improvement after 6 months of functional training. SPPB values of the ACEi group were significantly lower at the beginning of the study; however, we observed no difference between the SPPB results of the two groups after the training period. We conducted further studies to measure posture and spine mobility. Our Schober and Cobra test results revealed significantly improved spine mobility (both flexor and extensor) in both groups after 6 months of training. Furthermore, the grip strength of the hands, studied by an electric dynamometer, was significantly improved in both groups at the end of the training period. Our results indicated that functional training may improve body composition and muscle strength in patients diagnosed with sarcopenia. Furthermore, ACEi may be a helpful additional therapy in older adult patients suffering from severe sarcopenia.

## 1. Introduction

Sarcopenia is an age-related pathophysiological muscle atrophy. A pathological loss in muscle mass characterizes sarcopenia, leading to reduced mobility and quality of life [1]. The prevalence of sarcopenia is high and keeps on increasing. Today, an estimated 50 million individuals are affected globally, which will exceed 200 million by 2050 [2]. The underlying mechanisms of sarcopenia include hormonal alterations, immunological factors, nutritional deficiencies, and decreased physical activity. However, proper and early diagnosis and appropriate care and treatments may ease or even improve the disease symptoms. In addition to nutritional and exercise therapies, pharmacotherapeutic approaches may also play a role in treating sarcopenia. However, the literature is controversial regarding the effects of angiotensin-converting enzyme inhibitors (ACEi) on the physical performance of older patients.

The activity of the renin-angiotensin-aldosterone system contributes to skeletal muscle dysfunction through several pathways. Angiotensin II inhibits endothelial function and, consequently, muscle blood supply; moreover, it induces inflammation and increases the suppression of insulin-like growth factor 1 (IGF-1) and significantly reduces mitochondrial function. ACEi may have several potentially beneficial effects on skeletal muscle function. ACEi improve endothelial function and reduce inflammation. They enhance mitochondrial function; increase IGF-1 levels; promote skeletal muscle glucose uptake; suppress inflammatory cytokine levels, such as IL-6; and affect skeletal muscle function [3].

The Berlin Aging Study-II found no relationship between the muscle mass of patients and ACEi treatment. Spira et al. found no significant difference in muscle strength and muscle function upon ACEi administration [4]. Nevertheless, a recent trial has reported that perindopril significantly improved physical performance in seniors. Researchers conducted a 6-min walking test, and the total distance covered increased by 31 m [5]. Moreover, observational studies have found that patients taking ACEi have enhanced muscle strength and lower limb muscle mass [6].

Regarding pharmacotherapeutic approaches to sarcopenia, meta-analyses focused on 10 pharmacological interventions: vitamin D, pioglitazone combined, oestrogen-progesterone, dehydroepiandrosterone, growth hormone, growth hormone-releasing hormone, growth hormone combined with testosterone, insulin-like growth factor1-, testosterone, and ACEi. However, former studies have a significant limitation since they did not detail the severity of sarcopenia at baseline. Their recommendations are generalized to the elderly without determining whether the effects of drugs on muscle are more effective in healthy, presarcopenic, or sarcopenic older adults. Vitamin D significantly affected muscle strength and physical performance, especially in women with low baseline values (<25 nmol/L); side effects were rare. De Spiegeleer et al. conducted a study on men with low serum testosterone levels [7]. They suggested that testosterone intake significantly increased muscle mass but had a moderate or minimal impact on muscle strength and physical performance. They observed no significant side effects. Thus far, there is insufficient evidence to recommend other pharmacological interventions for improving sarcopenia.

In our study, we investigated the effect of a 6-month, regular functional exercise training program on patients with sarcopenia.

## 2. Materials and Methods

### 2.1. Subjects

A total of 36 female participants with mild to moderate sarcopenia were enrolled in the study. The criteria of sarcopenia diagnosis were based on the presence of low muscle function (low physical performance or low muscle strength) and low muscle mass according to the consensus of the European Working Group on Sarcopenia in Older People [2]. The first group (ACEi group) with normal blood pressure was prescribed ACE inhibitors 6 months before the investigation. Patients in the second (control) group did not receive ACEi treatment. One patient in the second control group was excluded from the study. Thus, 18 patients in the ACEi group (mean age: 66.17 ± 1.18 years) and 17 patients in the control group (mean age: 66.55 ± 1.29 years) completed the study. Participants enrolled in the investigation were nonsmokers, and they were abstaining from any physical exercises or sports activities, special diet, and vitamin supplements for at least 3 months prior to the study. Exclusion criteria included low plasma vitamin D level (<75 umol/L), statin therapy, oophorectomy, low protein diet, and decreased age-corrected glomerular filtration rate (GFR), as well as ongoing viral or bacterial infection, allergic or autoimmune disease, chronic disease treated with continuous drug therapy, cancer, alcohol or drug addiction, psychiatric illness, insufficient compliance, and dietary changes or usage of dietary supplements during the study period. Participants of the present study were trained two times, 45 min per week, for 6 months. We investigated the effect of functional training on sarcopenia.

### 2.2. Functional Training Program

The primary benefit of functional training is based on total body workout. The entire muscle chain is involved instead of selected muscles. Unstable devices may improve balancing stability. We incorporated aerobic exercises with a treadmill (Star Trac Model 8TRx, Core Health & Fitness LLC, Vancouver, NE, USA), elliptical trainer (Star Trac Model 8CT, Core Health & Fitness LLC, Vancouver, WA, USA), stationary bicycle (Activate Series Recumbent Lifecycle^®^ Exercise Bike, Life Fitness—Brunswick Corporation, Lake Forest, IL, USA), total body resistance exercise system (TRX) (TRX PRO4 System, Fitness Anywhere LLC, San Francisco, CA, USA), and unstable balls (Fitball, HPMS Inc., Windham, NH, USA) into our exercise program.

TRX suspension training enhances muscle strength, balance, and flexibility by using one’s body weight. TRX exercises improve the muscular strength of the forehand and small muscles of the hands. In older adult patients, the lower portion of the trapezius muscle is weakened, enhancing lumbar lordosis and the development of preplaced head position. The line of gravity in the human body is shifts forward, increasing the risk of falling. Pulling exercises with the TRX effectively strengthen the lower part of the trapezius muscle. TRX training improves the balancing ability and functional mobility in the elderly population [8]. During the execution of TRX exercises, the transverse abdominal muscle is continuously activated. This muscle is connected to the multifidus muscle via the thoracolumbar fascia. These two muscles play a crucial role in the stabilization of the lumbar portion of the spinal cord. Without sufficient core stability, intervertebral discs are overloaded, and the small intervertebral joints may be degraded. Heavy weight lifting exercises to increase muscle mass may overload joints and tendons. Training methods in elderly patients with comorbidities should be safe, and special attention needs to be given to joint protection. We excluded jumping exercises due to the higher risk of arthrosis.

An unstable ball is a valuable tool to enhance balancing ability. Proprioceptive exercises may support the adaptation of the neuromuscular system. These exercises may improve balancing ability and play a vital role in treating chronic back pain [9].

The exercise routine started with a 15 min warmup session. The participants completed low-intensity walking exercises; we gradually increased the intensity up to 50% of maximum heart rate (HRmax) to protect the joints. In the last 3 months, we increased the intensity up to 55% of HRmax. The first session included warming up large muscle groups of the upper and lower limbs. After the warmup session, we used the TRX to improve the strength endurance of the large muscle groups of the upper, lower limb, and core muscles. In the first 3 months, participants executed the TRX squat, TRX low rows, TRX push up, and TRX standing hip drop exercises. We gradually increased the intensity during the second 3-month period; TRX single leg squat, TRX single arm low rows, and TRX push up on one leg exercises were performed. We individually set the intensity (inclination angle) to allow the participants to execute 12 repetitions. Two participants performed the exercises in pairs, providing sufficient resting time.

The secondary purpose of our exercise program was to improve balancing ability. In the first 3 months of balance training, participants performed Fitball exercises with professional aid, later individually. Furthermore, exercises were performed with a light weight while sitting on a fitball in an imbalanced position to further strengthen upper limb muscle and enhance balancing.

All training was finished with a 10 min long stretching and relaxing session.

A summary of the training program is shown in Table 1.

### 2.3. Body Composition Assessment

We evaluated anthropometric and body composition data at the beginning and the end of the training program. Standard anthropometric measures, such as body weight, height, and body mass index (BMI), were measured and calculated. However, BMI is inadequate to measure the sarcopenia-related alterations; body fat ratio may distort the results.

Bioelectrical impedance analysis (BIA) is an up-to-date method to assess body composition thoroughly. We used the InBody 770 body composition analyser device (InBody, Cerritos, CA, USA) to measure body composition.

### 2.4. Short Physical Performance Battery Test

The Short Physical Performance Battery (SPPB) test is a validated method to measure the severity of sarcopenia. SPPB consists of a few simple tests enabling the disability of elderly patients to be predicted. Altered walking speed is a reliable indicator for decreased mobility and enhanced mortality [2,10]. Furthermore, the altered static balance may serve as an indicator for mobility and body control. Rising from the chair provides information about strength, balance, coordination, and mobility of the lower limb joints.

### 2.5. Handgrip Strength Measurement

Handgrip strength is an indicator of sarcopenia [11]. We utilized a Camry digital hand dynamometer (Camry Scale, South El Monte, CA, USA) for this purpose; values were given in kilograms. We conducted the measurements at the beginning and after completing the training program.

### 2.6. Posture Characterization Delmas Index

We used the Delmas index to assess the curve of the spinal cord. The Delmas index is the ratio of the actual length of the spinal cord divided by the extended length of the spinal cord multiplied by 100. Based on the results, we divided the patients into three groups. We set the normal range between 94 and 96. An index lower than 94 characterized so-called dynamic-type participants with a greater spinal cord curve than normal, while 96 and above were considered static types [11].

### 2.7. Cobra Test

The Cobra test aims to examine active mobility of the lumbar and dorsal spine. During the test session, the subject lies on a flat surface on the abdomen, and hands are placed under the shoulders to support. From this position, participants should push themselves so that the upper arm is in a vertical position and the pelvic region should remain on the mattress. At the maximum position performed, the distance of the incisura jugularis from the support should be measured at the maximum completed position [12].

### 2.8. Occiput Wall Distance Test

The purpose of this test was to assess the degree of deformity in the thoracic and cervical spinal cord area (kyphosis). Participants were asked to stand in an upright position against the wall. The heels, the buttock, and scapulae had to be in contact with the wall. Generally, participants should have been able to touch the wall with the occiput [13].

### 2.9. Schober Test

This test was developed to determine the range of motion (flexion) of the lumbar spine. The participants held themselves in an upright position. The examiner marked the second sacral vertebrae spine by drawing a horizontal line. The examiner marked a second line 10 cm above the first one. Participants bent forward while standing straight, and the pelvis was placed in a central position. In this position, the distance between the two lines was remeasured. The physiological value of the displacement of the lumbar section is 7–8 cm; the mean displacement is 5 cm. Displacement below 5 cm indicates decreased lumbar flexion mobility [12].

### 2.10. Statistical Analyses

We analysed data with the GraphPad Prism 8 software (Graphpad Software, San Diego, USA). Data were presented in graphs where bars show the mean, and each data point connected with a line displays an individual subject. Values in the text are displayed as mean ± standard deviation (SD) or median (25–75th percentiles) according to the distribution of the dataset. We used the Kolmogorov–Smirnov and Shapiro–Wilk normality tests to assess the distribution of the data. In case of Gaussian distribution, a two-tail paired *t*-test was used, but if the data set differed from normal distribution, the Wilcoxon test was performed. The differences between baseline data were analysed using either an unpaired *t*-test or Mann–Whitney U test. Differences were considered statistically significant at *p* < 0.05.

The sample size was calculated based on expected change regarding the outcome of physical intervention measured by the SPPB score. A change between 0.6 and 1.75 points has been shown to be clinically relevant in a number of studies where health improvement was measured in an older adult population after executing resistance-type exercise training [14,15,16,17,18].

However, in our study, we enrolled healthy elderly women who were 4–11 years younger and underwent a more intensive training program; thus, greater differences could be expected at the end of the intervention period. In order to detect a 2.3 mean of difference and 3.2 SD of difference (effect size of Cohen’s d = 0.71) in the SPPB score between the baseline and post intervention result, we estimated enrolling at least 18 individuals in each group to obtain 80% power (1-β) and 5% significance level (α = 0.05, two-tailed) in a paired samples *t*-test. The sample size calculation was performed using G*Power 3.1.3 (University of Düsseldorf, Düsseldorf, Germany).

## 3. Results

### 3.1. The Effects of Six-Month Training Program on Body Weight and Body Mass Index (BMI) in Elderly Females with Sarcopenia

Based on our observations, there were no or only minimal changes in body weight and BMI of the investigated individuals after finishing the training program. Anthropometric data is presented in Table 2.

### 3.2. Six-Month Training Program Improves Muscle Mobility and Posture in Elderly Females with Sarcopenia

**Figure 1:**
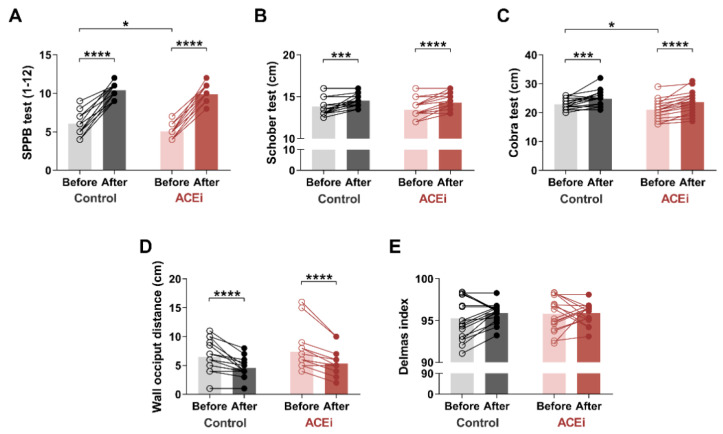
The effects of a 6-month functional training program on muscle mobility and posture in elderly females with sarcopenia. (**A**) Short Physical Performance Battery (SPPB) test. (**B**) Schober test. (**C**) Cobra test. (**D**) Occiput wall distance. (**E**) Delmas index. Bars show the mean, and each data point represents an individual subject. Statistically significant differences are defined as * *p* < 0.05; *** *p* < 0.001; **** *p* < 0.0001. Abbreviations: ACEi, angiotensin-converting enzyme inhibitors.

Based on SPPB measurements (Figure 1A) conducted on the ACEi group, two patients had moderate sarcopenia, while sixteen patients suffered from severe sarcopenia. However, after a 6-month exercise program, patients with severe sarcopenia demonstrated significant improvement: nine qualified for mild sarcopenia, and seven for the moderate stage. The two patients who previously had moderate sarcopenia demonstrated a mild stage of the disease by the end of the study. Regarding the control group, six patients had moderate stage, and twelve patients had a severe form of the disease based on the SPPB pretest. At the end of the 6-month exercise program, we were able to enrol four out of twelve patients with severe sarcopenia to the moderate stage. Seven patients demonstrated only mild symptoms. We upgraded six patients from the moderate sarcopenia to the mild sarcopenia group. Both groups demonstrated significant improvement in SPPB scores after completion of the 6-month exercise program (Control group: before vs. after: 6.0 (5.0–7.0) vs. 11.0 (9.5–11.0), respectively, *p* < 0.0001; ACEi group: before vs. after: 5.00 (4.00–5.25) vs. 10.00 (9.00–11.00), respectively, *p* < 0.0001). Our results indicated only a minor difference between the two groups (baseline SPPB in Control vs. ACEi groups: 6.0 (5.0–7.0) vs. 5.00 (4.00–5.25); *p* = 0.0248) by the end of the functional training program. Table 3 shows the component-specific before and after data of SPPB assessment.

We also assessed the flexion and extension mobility of the spine. Lumbar spine flexion was measured using the Schober test (Figure 1B). At the end of the exercise program, we observed significant improvement in both study groups (Control group: before vs. after: 13.5 cm (13.0–14.5) vs. 14.5 cm (14.0–15.25), respectively, *p* = 0.0005; ACEi group: before vs. after: 13.0 cm (13.0–14.0) vs. 14.0 cm (13.5–15.13), respectively, *p* < 0.0001).

The Cobra test was used to measure lumbar and dorsal spine extensions (Figure 1C). There was a significant improvement in both groups, with a greater improvement in the ACEi group (Control group: before vs. after: 22.88 ± 1.833 cm vs. 24.82 ± 2.698 cm, respectively, *p* = 0.0010 ACEi group: before vs. after: 21.06 ± 3.262 cm vs. 23.61 ± 3.775 cm, respectively, *p* < 0.0001). Furthermore, the initial difference between the two groups disappeared by the end of the functional training program (baseline values in Control vs. ACEi groups: 22.88 ± 1.833 cm vs. 21.06 ± 3.262 cm, respectively, *p* = 0.0495).

Based on the occiput wall distance test, the 6-month training significantly improved posture (cervical spine deformity) in both groups as well (Control group: before vs. after: 6.0 cm (5.0–8.75) vs. 4.0 cm (4.0–5.25), respectively, *p* < 0.0001; ACEi group: before vs. after: 6.0 cm (6.0–8.0) vs. 5.0 cm (4.0–6.0), respectively, *p* < 0.0001) (Figure 1D).

Regarding the Delmas index, the sagittal curves of the spine showed no significant change during the examination (Figure 1E). At the age of 65, we experience degenerative changes in the vertebrae, resulting in less mobilization of the spine for sagittal directions. Our 6-month physical activity program may hardly alleviate these symptoms.

### 3.3. Six-Month Training Program Enhances Muscle Mass and Function in Elderly Females with Sarcopenia

**Figure 2:**
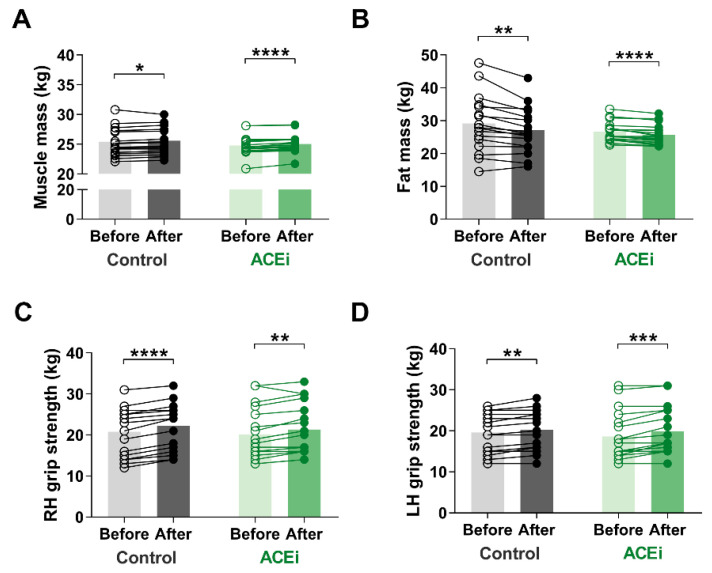
The effects of 6-month functional training program on muscle mass and function in elderly females with sarcopenia. (**A**) Muscle mass. (**B**) Fat mass. (**C**) Right hand (RH) grip strength. (**D**) Left hand (LH) grip strength. Statistically significant differences are defined as * *p* < 0.05; ** *p* < 0.01; *** *p* < 0.001; **** *p* < 0.0001.

Muscle mass is an important indicator of sarcopenia. In our study, muscle mass significantly increased in both groups due to the six-month functional training program. The improvement was more significant in those taking ACE inhibitors (Control group: before vs. after: 25.44 ± 2.365 kg vs. 25.59 ± 2.244 kg, respectively, *p* = 0.0300; ACEi group: before vs. after: 24.77 ± 1.635 kg vs. 25.03 ± 1.534 kg, respectively, *p* < 0.0001) (Figure 2A). Additionally, there was a significant decrease in fat mass in both groups by the end of the study (Control group: before vs. after: 29.16 ± 8.645 kg vs. 27.08 ± 6.950 kg, respectively, *p* = 0.0012; ACEi group: before vs. after: 26.67 ± 2.962 kg vs. 25.72 ± 2.977 kg, respectively, *p* < 0.0001) (Figure 2B). Regarding body composition, no significant difference was observed between the two groups.

We observed improvement on muscle mass and strength after completing the training program. The grip strength of both hands increased (Right hand grip: Control group: before vs. after: 23.0 kg (14.5–25.0) vs. 24.0 kg (16.5–26.0), respectively, *p* < 0.0001; ACEi group: before vs. after: 18.5 kg (15.75–25.5) vs. 20.5 kg (16.0–26.75), respectively, *p* = 0.0013; additionally, Left hand grip: Control group: before vs. after: 19.0 kg (15.0–24.5) vs. 20.0 kg (16.1–24.5), respectively, *p* = 0.0017; ACEi group: before vs. after: 16.0 kg (14.75–22.50) vs. 17.0 kg (15.75–25.00), respectively, *p* = 0.0002) (Figure 2B,C).

## 4. Discussion

Declining muscle function in older adult elderly patients increases the risk of falling and decreases their quality of life. In patients with sarcopenia, deterioration of muscle function is accelerated. Sarcopenia often leads to disability, loss of independence, and finally to death [19]. Accordingly, in a recent meta-analysis by Yeung et al., researchers found a higher risk for falls and fractures in patients with sarcopenia who had decreased muscle performance or reduced grip strength [20]. Balogun et al. investigated the relationship between lower handgrip strength and reduced muscle function as a predictor of falls and fracture [21].

Training programs and pharmacotherapy may be applied to improve muscle function, to enhance the quality of life, and to decrease the risk for disability. Our study underlined that functional training may effectively improve balance and muscle strength in the older adult population. Our results on body composition and grip strength indicated that muscle mass and strength improved significantly after completing our training program. Participants used their bodyweight and a training strap, and each participant could adjust their workout load accordingly. Changes in body fat data also supported our findings; moreover, we found that the functional training of women over 65 years of age may improve posture as well, which may prevent falling. Consequently, our training program might effectively reduce the risk of falls and related disabilities.

Additionally, our results supported our prior hypothesis that the difference in the severity of initial sarcopenia between ACEi and control groups may be alleviated by functional training. Furthermore, we found a more significant improvement in patients on ACEi therapy. Training programs in elderly patients may decelerate the physical decline in comparison with untrained counterparts [22]. However, pharmacotherapy may enhance the effect of training, supporting healthy aging [23]. Our results suggested that ACEi therapy could improve the beneficial effect of exercise in sarcopenic patients. Scientific evidence indicated that a decreased ACE level in young athletes with genotype II of the ACE gene is accompanied by higher endurance [24]. Decreasing ACE level by ACEi may contribute to a positive effect of training in the elderly. Taking ACE inhibitors may support muscle metabolism and physical therapy for older people with more severe sarcopenia. On the one hand, the metabolic effects of ACE inhibition, such as increased insulin sensitivity, glucose uptake, and glycogen storage, may improve muscle metabolic activity [25,26]. In addition, elevated bradykinin concentrations as a result of ACE inhibition may increase skeletal muscle blood supply, which may ultimately contribute to positive metabolic changes. As it was suggested earlier, this process may take a longer time [27]; thus, we have enrolled patients who were already on ACEi therapy at least for 6 months. In addition, ACE inhibition may reduce AT-II-associated inflammatory processes, which may also reduce age-related muscle loss [25]. This effect may be further enhanced by exercise therapy, as our previous results suggest that regular exercise improves immune regulation and immune response [28]. We hypothesized that the additional effect of ACEi therapy depends on the duration of intervention. Longer pharmacotherapy may be more beneficial for elderly patients. The results of Buford et al. supported our prior hypothesis. The authors found a better response to exercise programs in ACEi users than participants not taking antihypertensive medications [29]. However, we must mention some limitations of our research, namely that we only included female patients, the effects of social environment and cognitive functions were not evaluated, and changes in biomarker levels were not assessed in the study.

## 5. Conclusions

We may conclude that our training program is beneficial for patients suffering from different levels of sarcopenia. Our results suggested that treatment with ACEi for a more extended period (6 months in our study) may enhance the effect of physical training. However, to measure the exact time frame, further studies are required.

## Figures and Tables

**Table 1:** Training protocol (HRmax = maximal heart rate).

Schedule	Exercises	Intensity Level 1 (Month 1–3)	Intensity Level 2 (Month 4–6)
15 min	warm-up with treadmill/elliptic trainer/bicycle	50% HRmax	55% HRmax
20 min	TRX squat	Three sets of 12–15 reps	-
	TRX single leg squat	-	Three sets of 10 reps per foot
	TRX low rows	Three sets of 12–15 reps (range 10–45°)	-
	TRX single arm low rows	-	Three sets of 10 reps per arm (range 10–45°)
	TRX push up	Three sets of 12–15 reps (range 10–45°)	-
	TRX push up on one leg	-	Three sets of 10 reps per leg (range 10–45°)
	TRX standing hip drop	Three sets of 10 reps	Three sets of 15 reps
10 min	Fitball exercises	in pairs with professional aid	Individually
10 min	Stretching	within the normal range of motion	within the maximum range of motion

Abbreviations: HRmax, maximum heart rate; TRX, total body resistance exercise system.

**Table 2:** Anthropometric parameters of patients.

**Control Group**	**Before Training**	**After Training**	***p*** **-Values**
Body weight (kg)	72.09 ± 9.14	71.81 ± 8.76	n.s.
Height (cm)	163.35 ± 5.27	163.35 ± 5.27	n.s.
Body Mass Index (BMI)	26.96 ± 2.63	26.85 ± 2.45	n.s.
**ACEi-Treated Group**	**Before Training**	**After Training**	***p*** **-Values**
Body weight [kg]	71.08 ± 7.51	70.69 ± 7.27	0.014
Height [cm]	164.17 ± 6.34	164.17 ± 6.34	n.s.
BMI	26.33 ± 2.63	26.20 ± 1.70	0.029

Abbreviations: ACEi, angiotensin-converting enzyme inhibitors; n.s., not significant.

**Table 3:** Performance test scores for Short Physical Performance Battery (SPPB) assessment.

**Control Group**	**Before Training**	**After Training**	***p*** **-Values**
Standing balance (score)	2 (1.5–2.0)	4 (3.0–4.0)	<0.0001
Walk (score)	1 (1.0–2.0)	3 (3.0–3.5)	<0.0001
Chair stands (score)	3 (2.0–3.0)	4 (3.5–4.0)	0.0002
SPPB (score)	6 (5.0–7.0)	11 (9.5–11.0)	<0.0001
**ACEi-Treated Group**	**Before Training**	**After Training**	***p*** **-Values**
Standing balance [score]	1 (1.0–2.0)	3.5 (3.0–4.0)	<0.0001
Walk [score]	1 (1.0–2.0)	3 (2.0–4.0)	<0.0001
Chair stands [score]	2 (2.0–3.0)	3 (3.0–4.0)	<0.0001
SPPB [score]	5 (4.0–5.25)	10 (9.0–11.0)	<0.0001

## Data Availability

Datasets acquired for this study are included in the article. For further inquiries, contact the corresponding author.
